# Resveratrol Attenuates High-Fat Diet Induced Hepatic Lipid Homeostasis Disorder and Decreases m^6^A RNA Methylation

**DOI:** 10.3389/fphar.2020.568006

**Published:** 2020-12-18

**Authors:** Jiamin Wu, Yi Li, Jiayao Yu, Zhending Gan, Wenyao Wei, Chao Wang, Lili Zhang, Tian Wang, Xiang Zhong

**Affiliations:** College of Animal Science and Technology, Nanjing Agricultural University, Nanjing, China

**Keywords:** resveratrol, obesity, lipid metabolism, *N*^6^-methyladenosine RNA methylation, high-fat diet

## Abstract

**Purpose:** *N* ^6^-methyladenosine (m^6^A) mRNA methylation is affected by dietary factors and associated with lipid metabolism; however, whether the regulatory role of resveratrol in lipid metabolism is involved in m^6^A mRNA methylation remains unknown. Here, the objective of this study was to investigate the effect of resveratrol on hepatic lipid metabolism and m^6^A RNA methylation in the liver of mice.

**Methods:** A total of 24 male mice were randomly allocated to LFD (low-fat diet), LFDR (low-fat diet + resveratrol), HFD (high-fat diet), and HFDR (high-fat diet + resveratrol) groups for 12 weeks (*n* = 6/group). Final body weight of mice was measured before sacrificing. Perirhemtric fat, abdominal and epididymal fat, liver tissues, and serum were collected at sacrifice and analyzed. Briefly, mice phenotype, lipid metabolic index, and m^6^A modification in the liver were assessed.

**Results:** Compared to the HFD group, dietary resveratrol supplementation reduced the body weight and relative abdominal, epididymal, and perirhemtric fat weight in high-fat-exposed mice; however, resveratrol significantly increased average daily feed intake in mice given HFD. The amounts of serum low-density lipoprotein cholesterol (LDL), liver total cholesterol (TC), and triacylglycerol (TAG) were significantly decreased by resveratrol supplementation. In addition, resveratrol significantly enhanced the levels of peroxisome proliferator-activated receptor alpha (*PPARα*), peroxisome proliferator-activated receptor beta/delta (*PPARβ/δ*), cytochrome P450, family 4, subfamily a, polypeptide 10/14 (*CYP4A10*/*14*), acyl-CoA oxidase 1 (*ACOX1*), and fatty acid-binding protein 4 (*FABP4*) mRNA and inhibited acyl-CoA carboxylase (*ACC*) mRNA levels in the liver. Furthermore, the resveratrol in HFD increased the transcript levels of methyltransferase like 3 (*METTL3*), alkB homolog 5 (*ALKBH5*), fat mass and obesity associated protein (*FTO*), and YTH domain family 2 (*YTHDF2*), whereas it decreased the level of YTH domain family 3 (*YTHDF3*) and m^6^A abundance in mice liver.

**Conclusion:** The beneficial effect of resveratrol on lipid metabolism disorder under HFD may be due to decrease of m^6^A RNA methylation and increase of *PPARα* mRNA, providing mechanistic insights into the function of resveratrol in alleviating the disturbance of lipid metabolism in mice.

## Introduction

Lipids are critical nutrients and energy substances in both human and animals, whereas long-term high-fat diet (HFD) could result in defective nutritional metabolism, particularly in hepatic lipid metabolism (Feltenberger et al., 2013). Hepatic lipid metabolic disorder contributes to the development of obesity, which is involved in many serious chronic diseases, including diabetes, hypertension, and even cancer (Tessari et al., 2009). Therefore, further developing the effective investigation in the regulation of hepatic lipid metabolism is necessary and could offer potential theory to prevent and treat metabolic diseases.

Resveratrol (3,5,4′-trihydroxystilbene) is a natural polyphenolic compound found in plants. It is well known that resveratrol has antioxidative (Fu et al., 2018; Truong et al., 2018), anti-inflammatory (Xu et al., 2018; Zhou et al., 2018), anticarcinogenic (Kisková et al., 2014; Zheng et al., 2018), and antibacterial (Kukric and Topalic-Trivunovi, 2006; Duarte et al., 2015) effects and exhibits protective nature in the regulation of liver injury (Ajmo et al., 2008). Furthermore, accumulating evidence reported that resveratrol participates in attenuating abnormal lipid metabolism. Ran et al. (2017) found that the regulatory roles of resveratrol in lipid metabolism balance of zebrafish under dietary stress conditions are associated with the AMP-activated protein kinase alpha (AMPKα) pathway. Resveratrol also improves serum lipid characters and reverses body fat deposition in a pig model (Zhang et al., 2015). Sun et al. (2015) suggested that resveratrol could restore clock-mediated dysfunctional lipid metabolism in high-fat-fed mice via the activation of clock machinery. However, the potential molecular network of resveratrol in regulating lipid metabolism is unclear.

*N* ^6^-methyladenosine (m^6^A) is the most abundant mRNA modification in eukaryotes, which accounts for over 60% of all RNA chemical modifications (Wei et al., 1975). m^6^A modification can be dynamically installed, erased, and recognized by the m^6^A methyltransferase complex (METTL3, METTL14, and WTAP) (Liu et al., 2014; Ping et al., 2014; Wang et al., 2017), demethylases (FTO and ALKBH5) (Jia et al., 2011; Zheng et al., 2013), and m^6^A binding proteins (YTHDF1, YTHDF2, YTHDF3) (Dominissini et al., 2012; Wang X. et al., 2014; Liu et al., 2015). m^6^A RNA methylation has received great attention due to its function on cellular processes, including mRNA splicing, export, localization, translation, stability, and translation efficiency (Meyer et al., 2012; Liu et al., 2014; Wang Y. et al., 2014; Liu et al., 2015). In addition, m^6^A modification also plays a key role in biological processes such as cellular differentiation, lipid accumulation, and energy metabolism (Wang Y. et al., 2014; Zhao et al., 2014; Wang et al., 2015). Recently, dietary factors have been used to regulate m^6^A RNA methylation, such as betaine (Chen et al., 2015; Kang et al., 2018) and curcumin (Lu et al., 2018). Li et al. (2016) showed that maternal high-fat exposure led to imbalanced m^6^A mRNA modification in offspring. However, the effect of resveratrol on m^6^A modification is unknown.

We speculated that resveratrol in a HFD alleviated liver lipid metabolism disorders maybe due to the changes of m^6^A levels. Thus, the aim of this study was to investigate the effect of resveratrol on lipid metabolism and m^6^A RNA methylation in the liver of mice.

## Materials and Methods

### Animal and Diets

All experimental procedures were conducted in conformity with the Chinese Guidelines for Animal Welfare and were approved by the Animal Care Advisory Committee of Nanjing Agricultural University, China (NJAU-CAST-2015-095). Twenty-four C57BL/6J male mice (5 weeks of age) were from Yangzhou Institute of Experimental Animals [SCXK (Su) 2012-0004]. After 3 weeks of acclimation, mice were randomly distributed into four groups of six mice each as follows: 10% LFD and dietary supplemented with 276 mg/kg of resveratrol (LFDR), 60% HFD and dietary supplemented with 400 mg/kg of resveratrol (HFDR) (Kopec and Piatkowska 2013; Tomayko et al., 2014; Sun et al., 2015). There is 400 mg of resveratrol per kilogram of HFD, and the caloric value was about 5.2 kcal/g, while that of LFD was 3.6 kcal/g. In order to balance the amount of resveratrol per unit of energy between LFDR and HFDR diets, the amount of resveratrol per kilogram of LFD was 276 mg. During the entire 12-week experiment, all mice were housed at 22 ± 1°C under a 12-h light cycle and were allowed to drink and feed ad libitum. In addition, body weight and food consumption were recorded weekly.

Resveratrol (CAS: 501-36-0, purity over 99%) used in the experiment was bought from Sigma-Aldrich. We used high performance liquid chromatography (HPLC) analysis to confirm the concentration of resveratrol. All diets were manufactured by Trophic Animal Feed Co., Ltd. (Nantong, China). Composition and nutritional levels of mice diet were based on AIN93 (Reeves, 1997). The LFD group was fed a TP 2330055MC diet consisting of casein, starch, dextrin, sucrose, soybean oil, mineral mixtures, vitamin mixtures, cystine, choline, and tertiary butylhydroquinone (TBHQ). The HFD group was fed a TP 2330055M diet consisting of casein, starch, sucrose, lard, mineral mixtures, vitamin mixtures, cystine, choline, and TBHQ. The LFD consists of 10% fat, 14% protein, and 76% carbohydrate, and HFD consists of 60% fat, 14% protein, and 26% carbohydrate.

### Sample Collection

Mice body weight in the HFD group was higher up to 4 g (>4 g) than in the LFD group at the end of 12 weeks, suggesting that we successfully built a model of obesity (Hariri and Thibault 2010). Final body weight of mice was recorded before sacrificing. Peripheral blood samples were collected by cardiac puncture technique following anesthesia with carbon dioxide and centrifuged at 3,500 rpm/min for 10 min at 4°C after being kept in room temperature (RT) for 30 min, and the serum was collected from the supernatant of blood and stored at −80°C for the further determination. The liver was quickly removed, weighed, and thoroughly washed with phosphate buffer saline (PBS). A portion of the liver was stored separately in 10% buffered formalin solution for histopathological examination. The rest of the liver was snap-frozen using liquid nitrogen for further investigation. In addition, perirhemtric fat and abdominal and epididymal fat were immediately removed, weighed, and analyzed.

### Biochemical Parameters Analysis

The liver sample (0.2 g) from −80°C was suspended in ice-cold physiological saline (1.8 ml, 7.5 g/L NaCl diluent) and then homogenized at 13,500 *g* for 1 min in ice-bath using homogenizer (Tekmar, OH, United States). The homogenate was spun at 3,000 *g* for 15 min at a temperature of 4°C, and the supernatant was collected and analyzed immediately.

The levels of total cholesterol (TC, CAS: A111-1-1), triacylglycerol (TAG, CAS: A110-1-1), and low-density lipoprotein cholesterol (LDL, CAS: A113-1-1) were measured using commercial kits (Nanjing Jiancheng Bioengineering Institute, Jiangsu, China) by a microplate reader (Thermo Scientific, Wilmington, DE, United States) with a detection wavelength of 510, 510, and 546 nm, respectively. All experimental procedures were performed according to the manufacturer’s protocols. All hepatic measurements were normalized to concentrations of total protein for intersample comparisons.

### Hematoxylin and Eosin Staining

The liver sections fixed in 10% paraformaldehyde were dehydrated with graded dilutions of ethanol and embedded in paraffin. Then tissues (5 µm) were deparaffinized with xylene and rehydrated with graded dilutions of ethanol. The slides were stained with hematoxylin and eosin (H&E). A light microscope (Nikon ECLIPSE 80i, Nikon Corporation) was used to photograph and evaluate the pathological changes.

### Oil-Red Staining

For oil-red staining, fresh livers frozen at −80°C were sectioned (5 µm thick), fixed in a slide, and dissolved in propylene glycol (2 min). Slides were transferred to oil-red O solution (Sigma, Steindorf, Germany, CAS: O1516) for 1 h, then immersed in 85% propylene glycol (1 min), and washed two times in water. Finally, slides were counterstained in hematoxylin solution (10 s) and mounted using glycerin.

### RNA Extraction and Quantitative Real-time PCR

Total RNA of snap-frozen liver was extracted using TRIZol reagent (TaKaRa, Otsu, Shiga, Japan, CAS: 9108). The RNA integrity was examined on 1% of agarose gel using GelRed staining. The RNA contents were quantified by Thermo NanoDrop 2000 Ultra Trace Visible Spectrophotometer (Thermo Fisher, Waltham, MA, United States). After that, 1,000 ng total RNA was reverse-transcribed into cDNA in a 20 μl reaction volume using the PrimerScript RT Reagent kit (TaKaRa, Otsu, Shiga, Japan, CAS: RR036A). Real-time PCR was performed on the QuantStudioTM Design & Analysis Software (Thermo Fisher, Waltham, MA, United States). Primers were synthesized by Invitrogen Biotech Co., Ltd. (Shanghai, China) and listed in Table 1. qRT-PCR was performed in a 20 μl reaction mixture using ChamQ Universal SYBR qPCR Master Mix (Vazyme Biotech Co., Ltd., Nanjing, China, CAS: Q311-02). The thermal profile was 3 min at 95°C, 10 s at 95°C for 40 cycles, and then 30 s at 60°C. The relative gene expression was calculated based on the 2^−ΔΔCT^ method after normalization to housekeeping gene GAPDH. Samples in the LFD group were used as calibrator.

**TABLE 1:** Primers used for qRT-PCR.

Genes	Forward	Reverse
*GAPDH*	GGC​AAA​TTC​AAC​GGC​ACA​GT	AGA​TGG​TGA​TGG​GCT​TCC​C
*ACC*	GCC​TCC​GTC​AGC​TCA​GAT​AC	ATG​TGA​AAG​GCC​AAA​CCA​TC
*FABP4*	CTT​TGC​CAC​AAG​GAA​AGT​GG	TCC​CCA​TTT​ACG​CTG​ATG​AT
*FATP4*	ACT​GTT​CTC​CAA​GCT​AGT​GCT	GAT​GAA​GAC​CCG​GAT​GAA​ACG
*SREBP-1c*	GGA​GCC​ATG​GAT​TGC​ACA​TT	GGCCCGGGAAGTCACTGT
*PPaRγ*	CTGACAGGACTGTGTGAC	TCT​GTG​TCA​ACC​ATG​GTA​AT
*PPaRα*	TGC​AAA​CTT​GGA​CTT​GAA​CG	AGG​AGG​ACA​GCA​TCG​TGA​AG
*CYP4A10*	AGG​TGT​GGC​CAA​ATC​CAG​AG	AAT​GCA​GTT​CCT​GGC​TCC​TC
*CYP4A14*	ACC​CTC​CAG​CAT​TTC​CCA​TG	CTG​TAA​GCA​GGC​ACT​TGG​GA
*ACOX1*	CTG​GTG​GGT​GGT​ATG​GTG​TC	AAT​CTG​GCT​GCA​CGT​AGC​TT
*CPT1α*	GTG​AAA​AGC​ACC​AGC​ACC​TG	GAA​AGG​TGA​GTC​GAC​TGC​CA
*PPARβ/δ*	CCT​CCA​TCG​TCA​ACA​AAG​ACG	TTT​AGC​CAC​TGC​ATC​ATC​TGG​GCA​TGC​TC
*METTL3*	AGC​AGA​GCA​AGA​GAC​GAA​TTA​TC	GGT​GGA​AAG​AGT​CGA​TCA​GCA
*METTL14*	AGA​GAA​ACC​TGC​AGG​GCT​TC	TCC​TCC​TGC​TGC​ATT​TCC​AG
*FTO*	TTC​ATG​CTG​GAT​GAC​CTC​AAT​G	GCC​AAC​TGA​CAG​CGT​TCT​AAG
*ALKBH5*	CGC​GGT​CAT​CAA​CGA​CTA​CC	ATG​GGC​TTG​AAC​TGG​AAC​TTG
*YTHDF1*	ATG​CCC​AAC​CTA​CTT​CTG​CC	GAA​CAC​CCG​CCC​ACT​CTT​AA
*YTHDF2*	GAG​CAG​AGA​CCA​AAA​GGT​CAA​G	CTG​TGG​GCT​CAA​GTA​AGG​TTC
*YTHDF3*	ATG​CCC​AAC​CTA​CTT​CTG​CC	GAA​CAC​CCG​CCC​ACT​CTT​AA

*GAPDH*, glyceraldehyde-3-phosphate dehydrogenase; *ACC*, acyl-CoA carboxylase; *FABP4*, fatty acid-binding protein 4; *FATP4*, fatty acid transporter protein 4; *SREBP-1c*, sterol regulatory element binding protein-1c; *PPARα*, peroxisome proliferator-activated receptor alpha; *PPARγ*, peroxisome proliferator-activated receptor gamma; *CYP4A10*, cytochrome P450, family 4, subfamily a, polypeptide 10; *CYP4A14*, cytochrome P450, family 4, subfamily a, polypeptide 14; *ACOX1*, acyl-CoA oxidase 1; *CPT1α*, carnitine palmitoyltransferase 1 alpha; *PPARβ/δ*, peroxisome proliferator-activated receptor beta/delta; *METTL3*, methyltransferase like 3; *METTL14*, methyltransferase like 14; *FTO*, fat mass and obesity associated; *ALKBH5*, alkB homolog 5; *YTHDF1*, YTH domain family 1; *YTHDF2*, YTH domain family 2; *YTHDF3*, YTH domain family.

### Measurement of Total *N* ^6^-Methyladenosine

A total of 200 ng aliquots of mRNA was extracted from liver. EpiQuikTM m^6^A RNA Methylation Quantification Kit was used to detect total RNA m^6^A levels (Epigentek, Wuhan, China, CAT. No. p-9005) according to previous studies (Yuen et al., 2015; Slobodin et al., 2017; Zhang et al., 2017). Briefly, m^6^A on RNA was captured using m^6^A antibodies after binding to strip wells using binding solution. The signal of m^6^A was quantified colorimetrically via reading the absorbance on a microplate reader at 450 nm (Thermo Fisher, Waltham, MA, United States). The m^6^A level was calculated by OD intensity.

### Western Blot

Proteins from each 20 mg liver were extracted using tissue lysis buffer (Beyotime Biotechnology, Shanghai, China, CAS: P0013B) at a temperature of 4°C. Then, the homogenate was centrifuged at 12,000 *g* and 4°C for 30 min. The protein concentrations were measured using a commercial kit (Beyotime Biotechnology, Shanghai, China, CAS: P0012). Samples (40 μg of protein) were mixed with 5× sample buffer and boiled at 100°C for 10 min. Separation of the protein samples was performed on 10% sodium dodecyl sulfate polyacrylamide gel electrophoresis (SDS-PAGE) gels and electrotransferred onto the polyvinylidene fluoride (PVDF) membranes (Merck Millipore, Darmstadt, Germany, CAS: IPVH00010) with transfer buffer. The PVDF membranes were incubated overnight with primary antibody at a temperature of 4°C after being blocked in tris-buffered saline (TBS) containing 5% nonfat milk and 0.1% Tween-20 for 1 h at RT. After three times of washing, horseradish peroxidase-conjugated secondary antibodies (1:7,500, Abcam, ab205718, or ab205719) were applied to incubation of the membranes for 90 min at RT. The bands were visualized using enhanced chemiluminescence (ECL^Plus^) detection kit (Beyotime Biotechnology, Shanghai, China, CAS: P0018S). The images were analyzed by a luminescence image analyzer LAS-4000 system (Fujifilm Co., Ltd., Tokyo, Japan) and were quantified by Gel-Pro Analyzer 4.0 software (Media Cybernetics, Silver Spring, MD, United States). Some information about primary antibodies is as follows: methyltransferase like 3 (1:2,000, METTL3, Abcam, ab240595), YTH domain family 2 (1:2,000, YTHDF2, Proteintech, 24744-1-AP), alkB homolog 5 (1:1,500, ALKBH5, Proteintech, 16837-1-AP), FTO (1:1,500 Proteintech, 27226-1-AP), and β-actin (1:10,000, Proteintech, 60008-1-Ig).

### Statistical Analysis

Data were analyzed by the two-way analysis of variance (ANOVA) and were presented as means ± SD (standard deviations) after confirming normally distributed patterns. The classification variables were dietary resveratrol supplementation (LFD + HFD × LFDR + HFDR), HFD (LFD + LFDR × HFD + HFDR), and their interaction (LFD × LFDR × HFD × HFDR). The significant difference among groups was examined by Duncan’s multiple range tests when significant difference of resveratrol × HFD interaction was examined. The SPSS 21.0 statistical software (SPSS, Inc., Chicago, IL, United States) was used to analyze the present results. *p* values < 0.05 were considered as statistically significant level. *p* values < 0.01 were regarded as very significant.

## Results

### Effect of Resveratrol on Weight Gain and Feed Intake

**FIGURE 1:**
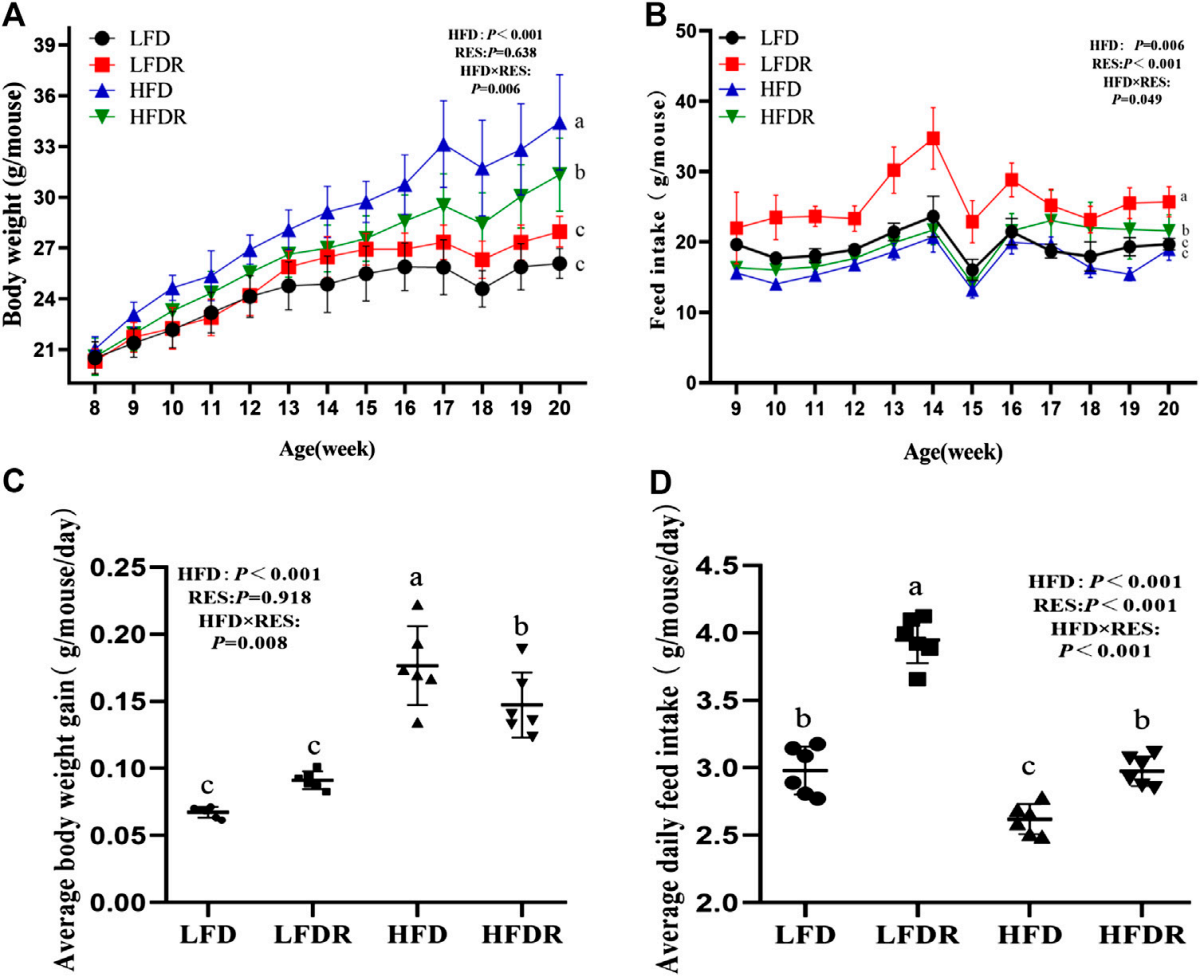
Resveratrol decreases body weight gain and enhances feed intake in HFD-treated mice. The body weight **(A)** and feed intake **(B)** were recorded every week, respectively. Average body weight gain **(C)** and average daily feed intake **(D)** were calculated. ^a–c^Different or the same superscript letters demonstrate statistically significant differences (*p* < 0.05) and no differences (*p* > 0.05) in groups, respectively (*n* = 6). LFD, LFDR, HFD, and HFDR represent low-fat, low-fat + 0.0276% resveratrol, high-fat, and high-fat + 0.04% resveratrol, respectively. HFD, high-fat diet; HFDR, high-fat diet + resveratrol; low-fat diet; LFDR, low-fat diet + resveratrol.

During the entire 12-week period, mice body weight in the HFD group was higher than that of the HFDR group (Figure 1A). The final body weight in mice fed LFD was significantly (32.87%) lower than mice in HFD exposure (*p* < 0.05, Figure 1A) and the food intake of mice in the LFDR group was the highest among the four experimental groups (*p* < 0.05, Figure 1B). We also found that resveratrol in a HFD significantly reduced the average daily gain in mice compared with the HFD group (*p* < 0.05, Figure 1C). Besides, the addition of resveratrol in HFD or LFD significantly increased the average daily feed intake (*p* < 0.05, Figure 1D). Intriguingly, a significant downward trend of feed intake was observed at 15 weeks. We speculated that this phenomenon may be attributed to differentiated hardness of feed between two individual packages (Sukemori et al., 2001).

### Effect of Resveratrol on Liver Weight and Fat Mass

**FIGURE 2:**
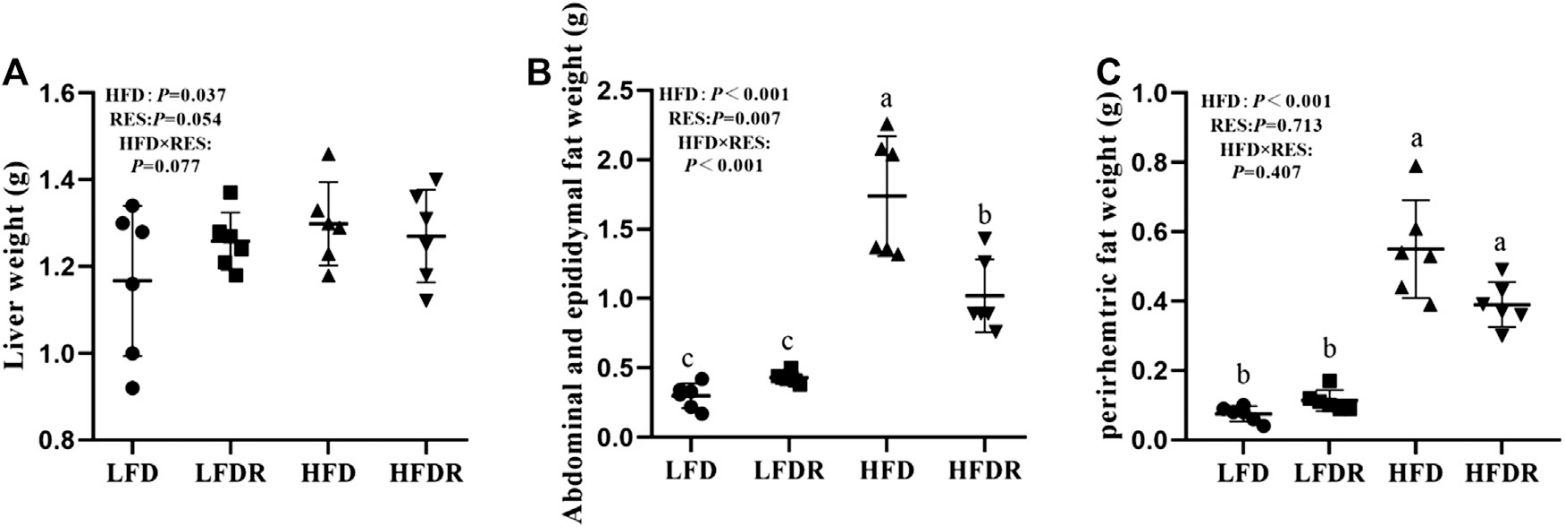
Resveratrol alleviates HFD induced fat mass. **(A)** Liver weight; **(B)** abdominal and epididymal fat weight; and **(C)** perirhemtric fat weight. ^a–c^Different or the same superscript letters demonstrate statistically significant differences (*p* < 0.05) and no differences (*p* > 0.05) in groups, respectively (*n* = 6). LFD, LFDR, HFD, and HFDR represent low-fat, low-fat + 0.0276% resveratrol, high-fat, and high-fat + 0.04% resveratrol, respectively. HFD, high-fat diet; HFDR, high-fat diet + resveratrol; low-fat diet; LFDR, low-fat diet + resveratrol.

Mice given HFD (HFD and HFDR) exhibited significant increase of the liver, abdominal, epididymal, and perirhemtric fat weight (*p* < 0.05, Figures 2A–C) relative to mice fed LFD (LFD and LFDR). Resveratrol significantly decreased the weight of abdominal and epididymal fat in the HFD compared to HFD group (*p* < 0.05, Figure 2B).

### Hepatic Morphology and Lipid Accumulation

**FIGURE 3:**
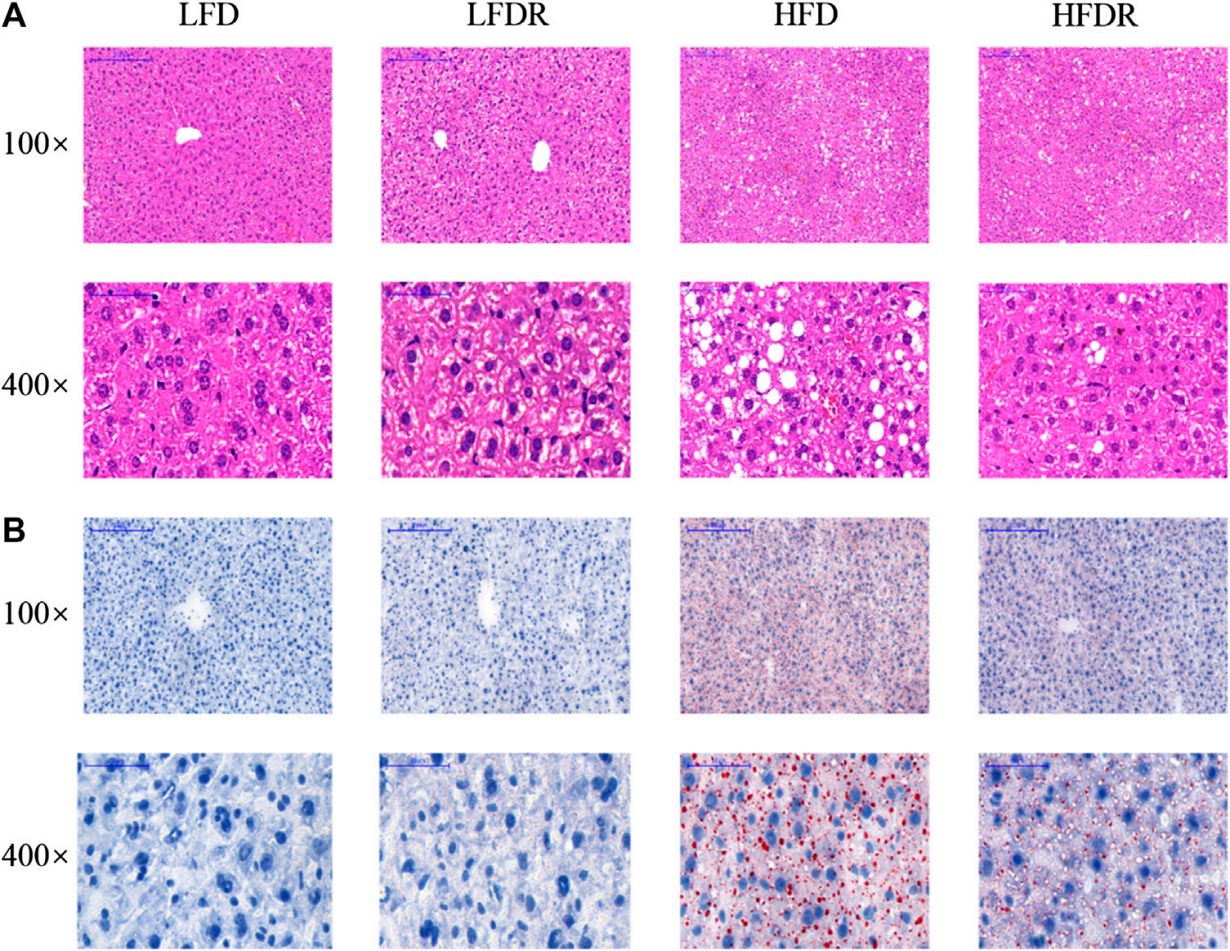
Resveratrol restores HFD induced abnormal lipid droplets in the liver. **(A)** Hematoxylin-eosin staining in the liver; and **(B)** oil-red O staining in the liver. Scale bar (100×) = 200 μm; scale bar (400×) = 50 μm. LFD, LFDR, HFD, and HFDR represent low-fat, low-fat + 0.0276% resveratrol, high-fat, and high-fat + 0.04% resveratrol, respectively. HFD, high-fat diet; HFDR, high-fat diet + resveratrol; low-fat diet; LFDR, low-fat diet + resveratrol.

Hepatic morphology and lipid accumulation were showed in Figure 3. Extensive macrocytic steatosis around the peripheral sinus region and fatty degeneration of microvesicles were observed in HFD mice. The steatosis and ballooning degeneration decreased by the addition of resveratrol in HFDR mice (Figure 3A). Further oil-red O staining analysis of those mice revealed the more appearance of lipid droplets within HFD group (Figure 3B). Moreover, treatment of resveratrol for 12 weeks decreased hepatic intracellular lipid droplets in HFDR mice.

### Lipid Metabolic Index

The contents of TC and LDL in the serum of HFD mice were significantly higher than those of LFD mice (*p* < 0.05, Table 2). The level of LDL was lower (*p* < 0.05) in serum of HFDR mice (*p* < 0.05) than HFD mice. However, the differences in TAG and TC in serum were not found between the HFD and HFDR groups. In addition, there was a significant enhancement (*p* < 0.05) in the concentrations of TAG, TC, and LDL in the liver of HFD mice compared with mice fed LFD alone. Notably, resveratrol could reverse the increase of TAG induced by HFD (*p* < 0.05).

**TABLE 2:** Resveratrol ameliorates HFD induced adverse lipid metabolism index in mice.

Items	LFD		LFDR		HFD		HFDR		*P* value		
	Mean	SD	Mean	SD	Mean	SD	Mean	SD	HFD	RES	HFD × RES
Serum											
TAG (mmol/L)	1.08	0.24	1.01	0.17	1.13	0.16	1.11	0.09	0.105	0.758	0.554
TC (mmol/L)	3.19^c^	0.36	3.69b^c^	0.43	4.40b^a^	0.42	4.28^ab^	0.83	0.001	0.410	0.173
LDL (mmol/L)	0.32^b^	0.06	0.33^b^	0.11	0.54^a^	0.29	0.35^b^	0.06	0.006	0.038	0.016
Liver											
TAG (mmol/gprot)	0.57^b^	0.07	0.65^b^	0.065	1.20^a^	0.122	0.70^b^	0.089	<0.001	0.012	0.001
TC (mmol/gprot)	2.40^b^	0.66	2.38^b^	0.47	3.44^a^	0.99	2.51^b^	0.41	0.046	0.130	0.152
LDL (mmol/gprot)	2.42^c^	0.53	2.84^bc^	0.60	4.23^a^	0.90	3.64^ab^	1.19	0.001	0.809	0.157

TC, total cholesterol; TAG, triacylglycerol; LDL, low-density lipoprotein; HFD, high-fat diet; HFDR, high-fat diet + resveratrol; low-fat diet; LFDR, low-fat diet + resveratrol. Different or the same superscript letters demonstrate statistically significant differences (*p* < 0.05) and no differences (*p* > 0.05) in groups, respectively (*n* = 6). LFD, LFDR, HFD, and HFDR represent low-fat, low-fat + 0.0276% resveratrol, high-fat, and high-fat + 0.04% resveratrol, respectively.

### Lipid Metabolism Associated Messenger RNA Expression

**FIGURE 4:**
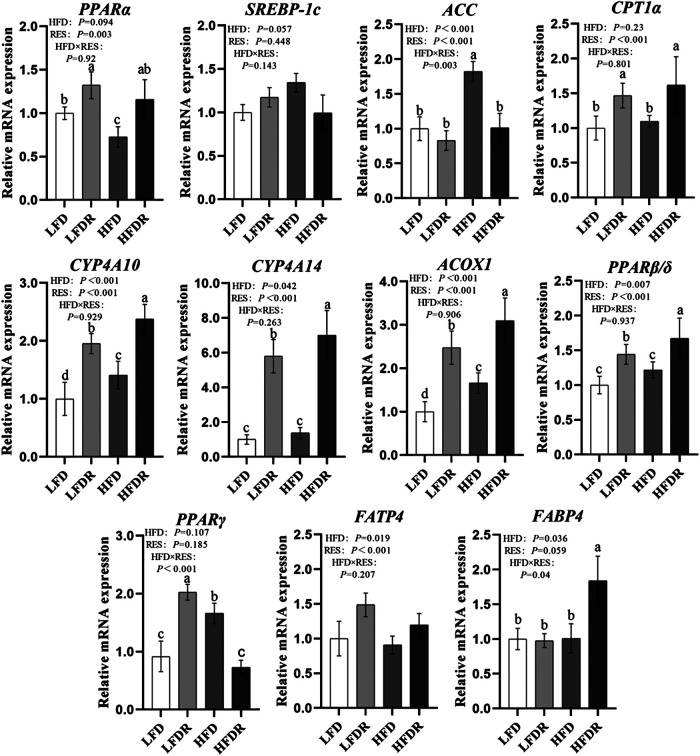
Resveratrol attenuates hepatic lipid metabolism disturbance in HFD-treated mice. ^a–d^Different or the same superscript letters demonstrate statistically significant differences (*p* < 0.05) and no differences (*p* > 0.05) in groups, respectively (*n* = 6). LFD, LFDR, HFD, and HFDR represent low-fat, low-fat + 0.0276% resveratrol, high-fat, and high-fat + 0.04% resveratrol, respectively. HFD, high-fat diet; HFDR, high-fat diet + resveratrol; low-fat diet; LFDR, low-fat diet + resveratrol.

We next measured the expression of lipid metabolism regulatory genes. Compared with LFD mice, HFD downregulated the expression of hepatic *PPARα* mRNA and upregulated the abundances of *ACC*, *SREBP-1c*, and *PPARγ* mRNA in the liver (*p* < 0.05, Figure 4). Dietary resveratrol supplementation in the HFD increased the expression of hepatic *PPARα*, *PPARβ/δ*, *CYP4A10*, *CYP4A14*, *ACOX1*, *FATP4*, and *FABP4* mRNA compared to mice given a HFD diet (*p* < 0.05). However, the levels of *ACC* and *PPARγ* mRNA in HFDR mice were reduced by resveratrol compared to untreated HFD group (*p* < 0.05). We also noted that dietary resveratrol in the LFD increased (*p* < 0.05) the expression of *PPARα*, *PPARβ/δ*, *CPT1α*, *CYP4A10*, *CYP4A14*, *ACOX1*, and *FATP4* mRNA relative to LFD.

### Effects of Resveratrol on *N* ^6^-Methyladenosine RNA Methylation

**FIGURE 5:**
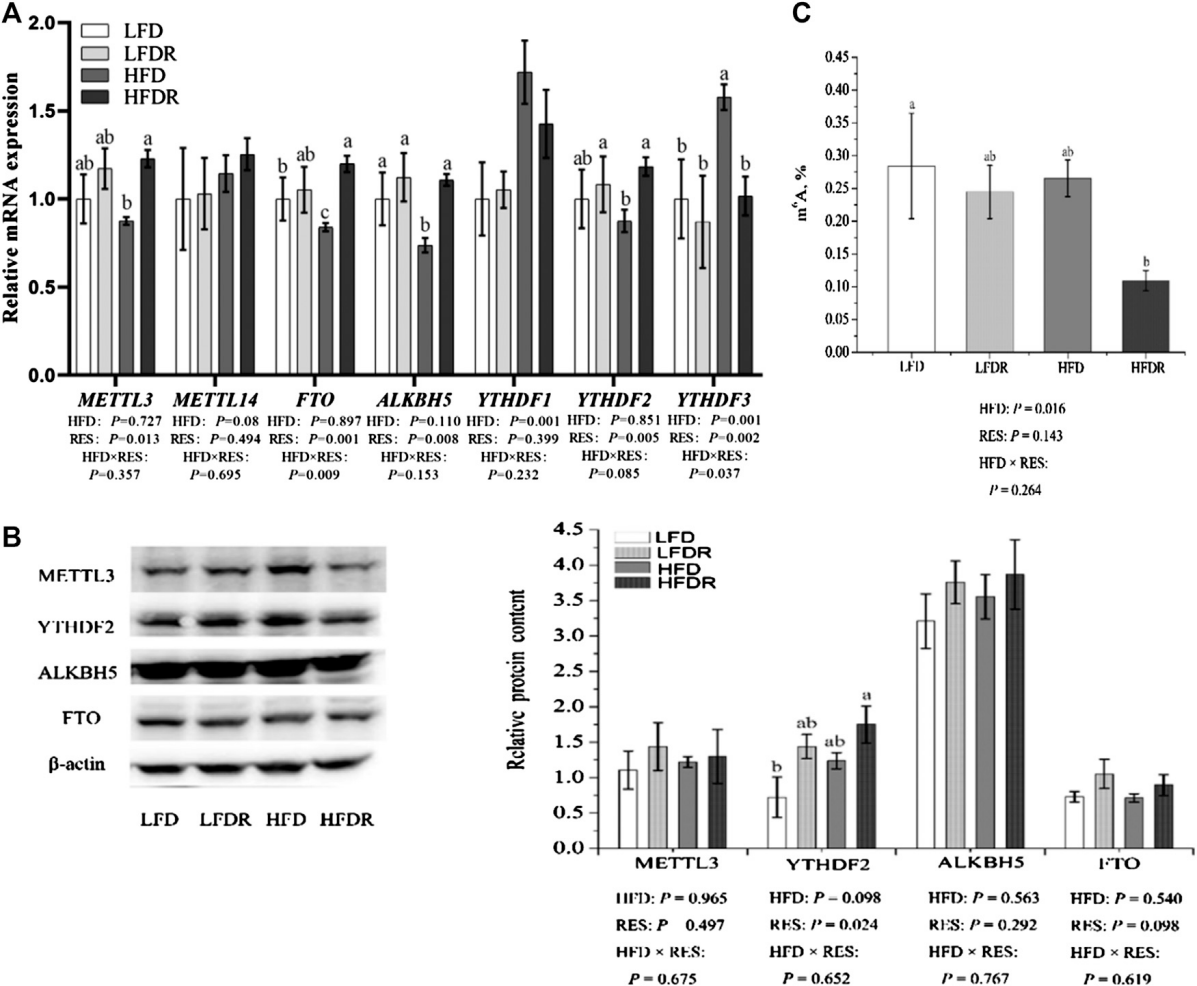
Resveratrol reduces the level of hepatic m^6^A RNA methylation in HFD-exposed mice. **(A)** m^6^A RNA methylation-related genes levels (*METTL3*, *METTL14*, *FTO*, *ALKBH5*, *YTHDF1*, *YTHDF2*, and *YTHDF3*); **(B)** m^6^A RNA methylation-related protein levels (METTL3, YTHDF2, ALKBH5, and FTO); and **(C)** the content of m^6^A. ^a–c^Different or the same superscript letters demonstrate statistically significant differences (*p* < 0.05) and no differences (*p* > 0.05) in groups, respectively (*n* = 6). (METTL3, 65–70 kDa; FTO, 58 kDa; ALKBH5, 40–50 kDa; YTHDF2, 62 kDa; β-actin, 42 kDa.) LFD, LFDR, HFD, and HFDR represent low-fat, low-fat + 0.0276% resveratrol, high-fat, and high-fat + 0.04% resveratrol, respectively. HFD, high-fat diet; HFDR, high-fat diet + resveratrol; low-fat diet; LFDR, low-fat diet + resveratrol; m^6^A, *N* ^6^-methyladenosine.

To investigate the regulation of resveratrol on mRNA m^6^A methylation, we tested m^6^A and m^6^A-related genes and proteins. Compared with LFD mice, HFD significantly downregulated the gene expression of *ALKBH5* and *FTO* while it obviously increased the level of *YTHDF3* (*p* < 0.05, Figure 5A). Moreover, resveratrol significantly elevated (*p* < 0.05) the levels of *METTL3*, *YTHDF2*, *FTO*, *ALKBH5*, and mRNA and decreased the mRNA expression of *YTHDF3* in HFDR mice (*p* < 0.05, Figure 5A). The results demonstrated that resveratrol significantly enhanced YTHDF2 protein (*p* < 0.05, Figure 5B). In addition, high-fat dietary resveratrol supplementation decreased the content of m^6^A (Figure 5C).

## Discussion

It is well known that HFD can induce obesity, abnormal lipid metabolism, and other relevant hepatic diseases. In this study, histologically, HFD induced obesity generated a typical and severe hepatic steatosis in mice, in accordance with a significantly increased phenotype of final body weight, accumulated concentration of hepatic TC and TAG, defective lipid droplets formed within hepatocytes, and disturbed transcriptional levels of lipid metabolism associated genes. Of note, growing evidence uncovers the protective potential of resveratrol in regulating lipid metabolism; however, its mechanism at the posttranscriptional level is still incompletely known. The current study provided evidence that dietary resveratrol supplementation protected against HFD induced aberrant lipid homeostasis and, as a result, restored hepatic steatosis and liver damage. Mechanistically, resveratrol intake facilitated the activation of PPARα and its downstream target genes at the transcriptional level and, meanwhile, decreased the m^6^A RNA methylation in the liver of mice. The present results, in part, indicate that the beneficial role of resveratrol in PPARα-dependent lipid metabolism may be involved in the modification of m^6^A RNA methylation.

PPARα, a molecular target of resveratrol (Takizawa et al., 2015), participates in the promotion of adipocyte differentiation, the modulation of nutritional metabolism, and the inhibition of inflammatory response (Kang et al., 2016; Brocker et al., 2017). Previous study showed that resveratrol increased the levels of sirtuin-1 (SIRT1) and PPARα to mediate its protective effect on hypertension under maternal HFD in the kidneys of male progeny (Tain et al., 2017). In addition, resveratrol enhanced hepatitis B virus transcription and replication followed by increase of transcriptional activity of PPARα in HepG2 cells and rats (Shi et al., 2016). Herein, hepatic elevation of *PPARα* mRNA was found in mice given resveratrol, together with the PPARα-dependent enhancement in expression of PPARα marker genes, including *CYP4A10*, *CYP4A14*, and *ACOX1*, which control the efficiency of fatty acid oxidation. So far, activation of PPARα transcription plays crucial roles in the regulation of resveratrol on lipid metabolism. Interestingly, growing investigations exhibited the interaction of PPARs with adipocyte-fatty acid-binding protein (A-FABP, FABP4), a late adipocyte differentiation marker. Fellous et al. recently confirmed that activation of PPAR receptors could promote the expression of FABP4 and other downstream genes (Fellous et al., 2020). Intriguingly, Boiteux et al. firstly found the positive correlation between FABP4 and PPARα in urothelial cancer cells (Boiteux et al., 2009). Likewise, Lu et al. also showed that increase of FABP4 expression was observed after activation of PPARα (Lu et al., 2004). The data presented herein confirmed the previous investigations and noted that resveratrol significantly increased the mRNA expression of *PPARα* and *FABP4* upon the exposure of HFD. Thus, we speculated that resveratrol-mediated PPARα activation regulated hepatic lipid metabolism by increasing the expression of FABP4. However, its potential mechanism at the epitranscriptomic level is not sufficiently known.

Accumulating evidence showed that m^6^A RNA methylation and hepatic lipid metabolic profile are closely intertwined. m^6^A takes place at transcriptional levels of nitrogen or oxygen atoms from S-adenosylmethionine (SAM) as a methyl donor (Niu et al., 2013). m^6^A can regulate mRNA splicing, export, localization, translation, and stability; thus it modulates the expression pattern of genes and proteins (Li et al., 2018; Yu et al., 2018). Liu et al. (2014) indicated that silence of METTL3 reduced the abundance of m^6^A and increased the transcriptional activity of PPARα in HeLa cells. Furthermore, our previous study discovered that reduction of m^6^A modification via silence of METTL3 or YTHDF2 upregulated the lifetime and expression of PPARα and affected the mRNA m^6^A methylation of PPARα and eventually reversed lipid accumulation (Zhong et al., 2018). As a result, these reports substantiated the robust regulatory role of m^6^A modification in PPARα-mediated lipid metabolic pathway. Of particular interest, it is worth noting that some dietary factors are sensitive to m^6^A methylation and metabolic regulation. The present study confirmed that resveratrol intake beneficially affects transcriptional levels of lipid metabolic genes, especially the activity of PPARα, and alleviates hepatic lipid accumulation together with elevation of mRNA levels of m^6^A methylases and demethylases, increase of YTHDF2 expression, and obvious reduction of *YTHDF3* mRNA expression and hepatic m^6^A level. For example, maternal HFD changes mRNA m^6^A modification and its regulatory genes in offspring (Li et al., 2016). In addition, cycloleucine (methylation inhibitor) and betaine (methyl donor) oppositely modulate m^6^A levels and lipid deposition (Kang et al., 2018). Moreover, our previous data also indicated that dietary curcumin or resveratrol supplementation changed the hepatic m^6^A abundance and affected liver function in piglets (Lu et al., 2018; Gan et al., 2019). Thus, these observations, in part, suggested that the protective role of resveratrol in maintaining lipid metabolism could attribute to the regulation of transcriptional PPARα activity and the modification of m^6^A methylated lipid metabolism-related genes. Further investigation is required to explore the precise crosstalk between resveratrol-regulated lipid homeostasis and m^6^A RNA methylation.

## Conclusion

Resveratrol attenuated HFD induced abnormal lipid metabolism and affected m^6^A profiles in the liver of mice. The alleviating effect of resveratrol on disorder of lipid metabolism under HFD may be associated with the decrease of m^6^A methylation and increase of *PPARα* mRNA. The present work offers insights into the underlying avenues for the treatment of some relevant liver diseases.

## Data Availability Statement

The raw data supporting the conclusions of this article will be made available by the authors, without undue reservation, to any qualified researcher.

## Ethics Statement

The animal study was reviewed and approved by the Animal Care Advisory Committee of Nanjing Agricultural University, China (NJAU-CAST-2015-095).

## Author Contributions

JW, YL, and XZ designed the research and wrote the paper, and JW was a major contributor in writing the manuscript. JW, YL, and ZG searched and read the literature. JW, YL, WW, and JY performed experiments. CW, LZ, and TW provided essential suggestion and revision. XZ had primary responsibility for final content. All authors read and approved the final manuscript.

## Funding

This study was supported by grants from the National Natural Science Foundation of China (No. 31872391).

## Conflict of Interest

The authors declare that the research was conducted in the absence of any commercial or financial relationships that could be construed as a potential conflict of interest.

## Glossary

ACC: acyl-CoA carboxylase
ACOX1: acyl-CoA oxidase 1
ALKBH5: alkB homolog 5
AMPKα: AMP-activated protein kinase alpha
ANOVA: analysis of variance
CPT1α: carnitine palmitoyltransferase 1 alpha
CYP4A10: cytochrome P450, family 4, subfamily a, polypeptide 10
CYP4A14: cytochrome P450, family 4, subfamily a, polypeptide 14
ECLPlus: enhanced chemiluminescence
FABP4: fatty acid-binding protein 4
FATP4: fatty acid transporter protein 4
FTO: fat mass and obesity associated
H&E: hematoxylin and eosin
HFD: high-fat diet
HFDR: high-fat diet + resveratrol
HPLC: high performance liquid chromatography
LDL: low-density lipoprotein cholesterol
LFD: low-fat diet
LFDR: low-fat diet + resveratrol
m6A: *N* ^6^-methyladenosine
METTL3: methyltransferase like 3
METTL14: methyltransferase like 14
PBS: phosphate buffer saline
PPARα: peroxisome proliferator-activated receptor alpha
PPARβ/δ: peroxisome proliferator-activated receptor beta/delta
PPARγ: peroxisome proliferator-activated receptor gamma
PVDF: polyvinylidene fluoride
RT: room temperature
SAM: S-adenosylmethionine
SD: standard deviations
SDS-PAGE: sodium dodecyl sulfate polyacrylamide gel electrophoresis
SIRT1: sirtuin-1
SREBP-1c: Sterol regulatory element binding protein-1c
TAG: triacylglycerol
TBHQ: tertiary butylhydroquinone
TBS: tris-buffered saline
TC: total cholesterol
WTAP: Wilms’ tumor 1-associating protein
YTHDF2: YTH domain family 2
YTHDF3: YTH domain family 3
